# Non diagnosed PAPVC induce large reverse venovenous shunt after modified Fontan surgery: A case report of a rare anomaly and embolization therapy

**DOI:** 10.34172/jcvtr.2021.22

**Published:** 2021-04-14

**Authors:** Zahra Alizadeh Sani, Abdolrahim Ghasemi, Shabnam Mohammadzadeh, Zahra Khajali, Mohaddeseh Behjati, Roohallah Alizadehsani, Abbas Khosravi, Saeid Nahavandi, Sheikh Mohammed Shariful Islam

**Affiliations:** ^1^MRI Department, Shaheed Rajaei Cardiovascular & Medical Research Center, Iran University of Medical Sciences, Tehran, Iran; ^2^Modarres Hospital, Shahid Beheshti University of Medical Sciences, Tehran, Iran; ^3^Imam Khomeini Hospital, Tehran University of Medical Sciences, Tehran, Iran; ^4^Shaheed Rajaei Cardiovascular & Medical Research Center, Iran University of Medical Sciences, Tehran, Iran; ^5^Institute for Intelligent Systems Research and Innovation, Deakin University, Geelong, VIC 3216, Australia; ^6^Institute for Physical Activity and Nutrition, Deakin University, Melbourne, Australia; ^7^Cardiovascular Division, The George Institute for Global Health, Australia; ^8^Sydney Medical School, University of Sydney, Australia

**Keywords:** Venovenous Collateral, Fontan, Cardiac Magnetic Resonance Imaging

## Abstract

Fontan operation is a reliable palliative surgery for patients with single ventricle physiology. Still, the development of complication is common; one of these complications that need to interventional approach is veno-venous collaterals between systemic and pulmonary veins. A 16-yearoldgirl with a history of modified Fontan operation at 9 years ago was referred with progressive cyanosis and dyspnea on exertion. In contrast trans-thoracic echocardiography (TTE), no fenestration was seen in Fontan circulation. Cardiac magnetic resonance revealed partial anomalous pulmonary vein connection (PAPVC) from left upper pulmonary vein to vertical vein and then into the in nominate vein and SVC with the reverse flow from superior vena cava (SVC) to left upper pulmonary vein(LUPV). This anomalous vein became severe engorged and tortuous. Possibly, LUPV and the verticalvein was dilated gradually as a result of increased pressure in the Fontan circuit. Finally, she underwent successful coil embolization in the midpart of the vertical vein. The oxygen saturation increased from80% to 93%.

## Introduction

Several modifications of Fontan procedure have been used as palliation of functionally univentricular circulation since the early 1970s. The most common cause of cyanosis in patients after Fontan is fenestration in the baffle. Other etiologies are shunting through fistulae, arteriovenous malformations (AVMs), and veno-venous collaterals. The development of this collaterals first reported in 1998. These collaterals are common between superior vena cava (SVC) or innominate vein, but also can develop in hepatic veins. This report is in accordance with human research recommendation of Iran University of Medical Sciences Ethics Committee.

## Case Presentation

A 16-year-old girl referred us with progressive cyanosis and dyspnea on exertion (functional class II, O_2_ saturation at room air = 80%). In her history, single ventricle, D-malposition of great arteries, pulmonary atresia, patent ductus arteriosus (PDA) and straddling of tricuspid valve were revealed. She underwent bidirectional Glenn shunt on March 2005, at her 4th years of age. On February 2007, at 6 years of age, she underwent extracardiac Fontan operation, aortic valve (AV) repair, atrial septectomy and pulmonary artery reconstruction. Nine years following the operation, the patient developed progressive cyanosis and dyspnea. At this time, physical examination revealed mild peripheral cyanosis and clubbing. Heart auscultation was unremarkable.

**Figure 1:**
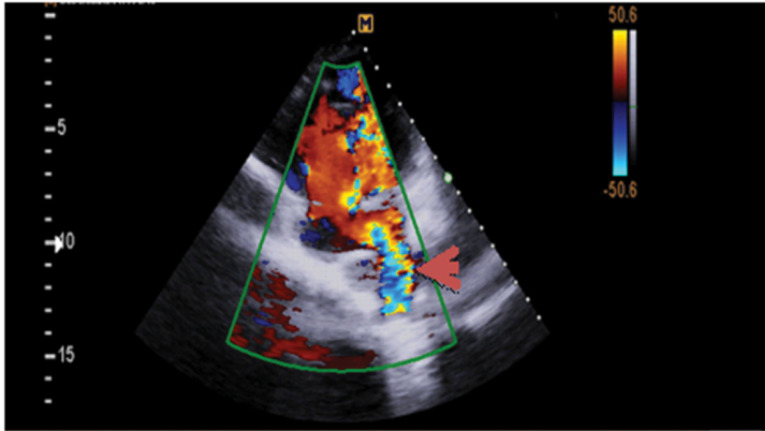
Color doppler flow in trans- thoracic echocardiography shows turbulent flow from left inferior pulmonary vein into left atrium (red arrow)

**Figure 2:**
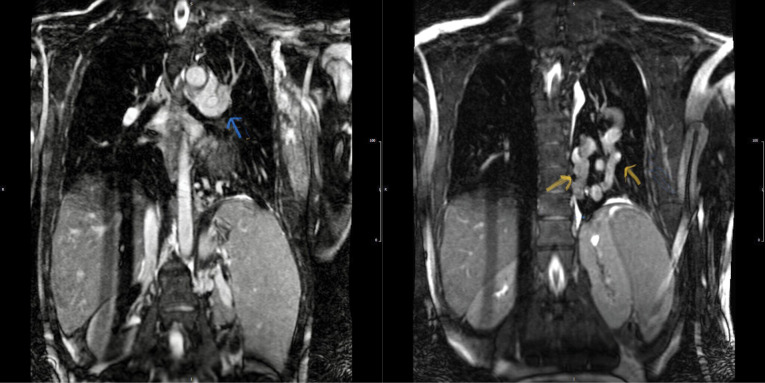
Magnetic resonance angiography imaging (A)left upper pulmonary vein connection into the vertical vein (blue arrow) (B) tortuous and the large fistulous connection between left upper and lower pulmonary vein (yellow arrows)

**Figure 3:**
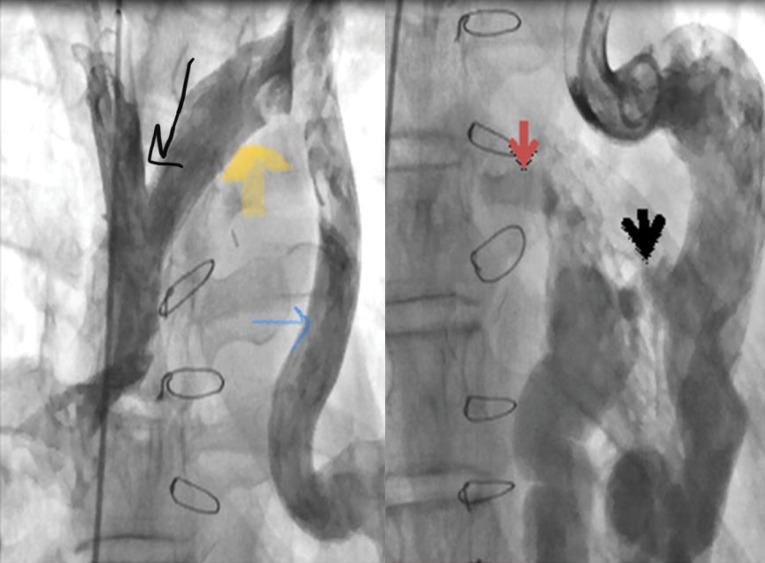
Cardiac catheterization disclosed reflux of contrast from the vertical vein into the innominate vein and superior vena cava(A).A large feeder vessel arises from the left vertical vein with fistulous connection to the left inferior pulmonary vein which drained into left atrium (red arrow). A plexus of veins arising from the descending limb of fistulous connection and drained immediately into ascending limb of Venous fistula (thick black arrow)(B).

**Figure 4:**
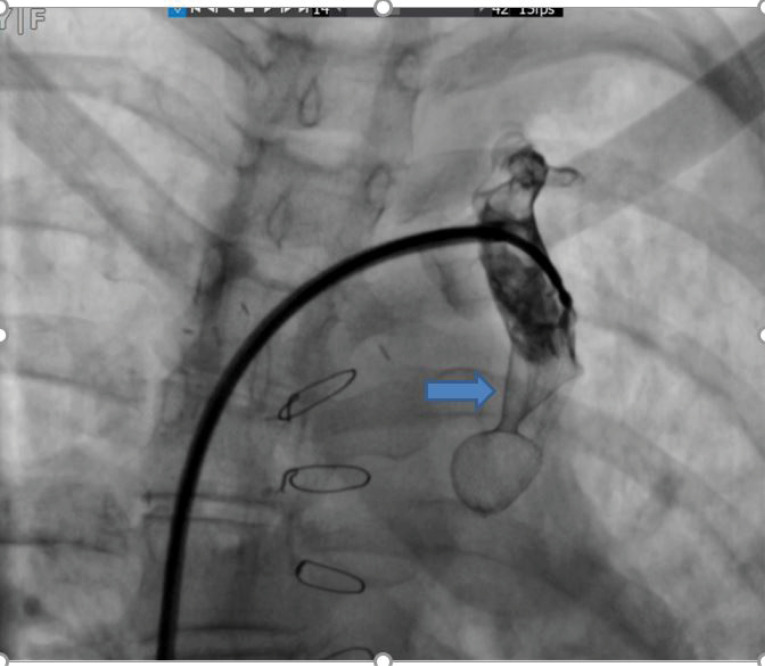
ADO device at proximal of vertical vein

Hemoglobin concentration was 17g/dL. Electrocardiogram showed low voltage, poor R progression and left anterior hemiblock. Contrast trans-thoracic echocardiography (TTE) with an injection of agitated saline from left brachial vein showed a left-sided dilated para-aortic vessel including a blue color descending flow suggested left superior vena cava (LSVC) with a fast entrance of bubbles into the left atrium (Figure 1). Finally, she underwent cardiac magnetic resonance imaging (CMR) due to cyanosis which revealed the presence of very engorged dilated left lower and left upper pulmonary veins with fistulous connection which left upper pulmonary vein (LUPV) connected to vertical vein and then into an innominate vein and finally SVC. The direction of flow was from SVC to left upper pulmonary vein, left lower pulmonary vein, and left atrium (LA). (Figure 2A, 2B). This Fistula was seen as collateral vessels connected proximally to the inferior aspect of innominate vein and distally to the left lower pulmonary vein. There were some connections between the ascending, and descending limbs of these tortuous venous collaterals and these direct connections between venous collateral vessels could explain fast pass of agitated saline into LA during TTE with contrast study and severe cyanosis of the patient. Probably this abnormal connection of LUPV to vertical vein (PAPVC) was not diagnosed before Fontan operation and developed gradually due to the pressure gradient in the Fontan circuit with connection to LLPV. This pressure gradient could result in further dilatation of vertical vein and prominence of collateral vessels which were connected to the inferior aspect of innominate vein. Thus, catheterization was performed (Figure 3A and 3B) and Femoral vein was cannulated. We passed from IVC to fontan circuit, SVC, innominate and lastly vertical vein. In injection in vertical vein, we found severe engorged and tortoise pulmonary veins, then a long stiff exchange wire (260 cm) with a long LIFETECH delivery sheath 9F passed through fontan circuit to SVC, innominate vein and vertical vein. An ADO device 14*16 mm chosen and deployed at proximal of vertical vein (Figure 4). After the detachment of device by vertical injection we confirmed proper position of device and small residual flow. The oxygen saturation immediately increased from 80% to 93%. In the first echocardiography performed after device closure, some contrast agents were entered into the left atrium through a right to left shunt, which indicates the presence of a residual shunt. This might be due to the delayed endothelialization of implanted device.

## Discussion

A significant part of the patients with long-standing cavo- pulmonary shunts are at increased risk for hypoxia due to right to left shunt via the development of pulmonary arteriovenous malformation (PAVM), venovenous malformation and various forms of vascular malformations.^1^ PVAM is more common compared veno-venous collaterals, and this is as a result of low-pressure flow, loss of pulsatile circulation and absence of hepatic factor.

The development of veno-venous collaterals is due to elevated pressure in Fontan circulation that resulted in the reestablishment of remnant embryologic connection between the systemic venous to pulmonary venous system.^2^ Our case is one of the rare anomalies in these type of collaterals that non-diagnosed PAPVC was the potential connection between the systemic venous and pulmonary venous systems which after the Fontan operation this connection developed and flow became reverse from systemic vein to pulmonary vein system. The first image modality for evaluation of these vascular malformations is considered to be contrast echocardiography, but in some instances, it fails. One of the best modality imaging in Fontan patients is CMR meets the exact anatomic and also hemodynamic evaluation, and it can calculate the degree of right to left shunt via veno-venous collaterals.^3^

These abnormal connection needs to be closed and in the most situation, this procedure performed via percutaneous catheter intervention. The first reports showed occlusion by coil embolization and ductal occluders, but nowadays vascular plugs and septal occluders have been used for this purpose.

## Conclusion

Probing the cause of hypoxia in Fontan patients is always essential. CMR is a safe and non-invasive method in this regard. Our reported case is the unique case with non-diagnosed PAPVC before the operation that in this abnormal connection, the flow direction became reverse after surgery, and the all venous connection became engorged and dilated.

## Competing interest

The authors stated that they had no conflict of interest.

## Ethical approval

None.

## Funding

This study was not funded or supported by any government or non-government organizations.
